# Effect of Optimized UV-LED Technology on Modeling, Inactivation Kinetics and Microbiological Safety in Tomato Juice

**DOI:** 10.3390/foods13030430

**Published:** 2024-01-29

**Authors:** Fernando Salazar, Sebastián Pizarro-Oteíza, Sebastián Molinett, Mariela Labbé

**Affiliations:** 1 Laboratorio de Fermentaciones Industriales, Escuela de Alimentos, Facultad de Ciencias Agronómicas y de los Alimentos, Pontificia Universidad Católica de Valparaíso, Av. Waddington 716, Valparaíso 2340000, Chile; 2 Laboratorio de Bionanotecnología, Instituto de Investigaciones Agropecuarias, INIA CRI La Cruz, Chorrillos 86, La Cruz 2280454, Chile

**Keywords:** UV-LED irradiation, tomato juice, *Escherichia coli* O157:H7, *Listeria monocytogenes*, Weibull modeling, microbiological safety

## Abstract

This research analyzed, optimized and modeled the inactivation kinetics of pathogenic bacteria (PB1: *Escherichia coli* O157:H7 and PB2: *Listeria monocytogenes*) and determined the microbiological safety of tomato juice processed by UV-LED irradiation and heat treatment. UV-LED processing conditions were optimized using response surface methodology (RSM) and were 90% power intensity, 21 min and 273–275 nm (251 mJ/cm^2^) with R^2^ > 0.96. Using the optimal conditions, levels of PB1 and PB2 resulted a log reduction of 2.89 and 2.74 CFU/mL, respectively. The Weibull model was efficient for estimating the log inactivation of PB1 and PB2 (CFU/mL). The kinetic parameter δ showed that 465.2 mJ/cm^2^ is needed to achieve a 90% log (CFU/mL) reduction in PB1 and 511.3 mJ/cm^2^ for PB2. With respect to the scale parameter *p* > 1, there is a descending concave curve. UV-LED-treated tomato juice had an 11.4% lower *Listeria monocytogenes* count than heat-treated juice on day 28 (4.0 ± 0.82 °C). Therefore, UV-LED technology could be used to inactivate *Escherichia coli* O157:H7 and *Listeria monocytogenes*, preserving tomato juice for microbiological safety, but studies are required to further improve the inactivation of these pathogens and analyze other fruit and vegetable juices.

## 1. Introduction

Fruit juices provide bioavailable and bioactive micronutrients at levels similar to those of whole fruit [1,2]. Particularly, tomato (*Solanum lycopersicum*) is an important food for human nutrition and is the second most important horticultural crop worldwide [3]. Tomato products have high nutritional value and functional properties derived from lycopene and polyphenols [4]. For this reason, the tomato industry maintains important challenges related to production quality. Andreou et al. [5] mentioned that pilot-scale processing of tomato products (e.g., juice, paste and sauce) includes thermal treatments such as heating and pasteurization to inactivate microorganisms and pectolytic enzymes, as well as to prolong shelf life [6,7]. Thermal processing ensures the safety and shelf life of fruit juices but contributes to the loss of functional and nutritional quality [8]. For this reason, non-thermal technologies have been undergoing exponential research with a focus on food preservation in recent years [9,10,11,12].

Ultraviolet (UV) light is one promising technology for inactivating microorganisms [13,14,15]. Fortuitously, UV light irradiation is one of the efficient methods for pathogen control [14,15]. However, for the correct use of UV light, it is important to define the regions of the electromagnetic spectrum. UV light occupies the electromagnetic spectrum region from 100–400 nm and, according to Koutchma [16], can be classified into UV-A (315–400 nm), UV-B (280–315 nm), UV-C (180–280 nm) and vacuum UV ranges (100–200 nm). Among the range of UV lights, UV-C has a germicidal effect on microorganisms such as yeasts, viruses, mold and bacteria [9,17,18]. Furthermore, Koutchma [16] mentioned that implementation of this process could be applicable to various food products such as fruit juices and vegetables because, in 2002, the Food and Drug Administration (FDA) approved UV light treatment on food surfaces and in fruit juices.

Ultraviolet light, as a non-thermal technology, has numerous mechanisms of action on micro-organisms depending on its type and wavelength that target a specific cellular component, thus causing some biological effects [19]. This is mainly due to the fact that, at this wavelength, the formation of a photoproduct (cyclobutane-pyrimidine) is generated, which interrupts Deoxyribonucleic Acid (DNA) translation and transcription [13,20,21]. In addition, Taze et al. [22] and Kebbi et al. [19] commented that incident UV light hits nucleic acids and its constituents absorb the light, resulting in mutations and cytotoxic lesions in DNA such as pyrimidine dimers (PD), which is an intrastrand cross-link, in which two adjacent pyrimidines are connected by a cyclobutane ring. Conceptually related, but structurally distinct, is the less frequent 6,4-photoproduct, which is also a pyrimidine dimer but without the cyclobutane ring [19].

On the basis of the above, Cadet et al. [23] and Ramesh et al. [24] described that another mechanism of DNA destruction in microorganisms occurs when UV irradiation generates Reactive Oxygen Species (ROS). Photosensitizers are substances naturally present in the microbial cell and are stimulated to a higher energy level state. To generate this stimulation, Prasad et al. [25] mentioned that ROS molecules (peroxides, oxygen and superoxide ions) play an important role. These molecules are formed when excited photosensitizers impact with adjacent cytoplasmic molecules and interact with lipids, proteins and DNA, and cause a cytotoxic effect in microorganisms.

Commonly, UV technology uses mercury lamps for food disinfection [26,27]. However, despite their germicidal efficacy, they have several disadvantages, such as contamination of food with mercury, fragile material, shelf life not more than 15,000 h and require pretreatment to reach their maximum optical power. Currently, UV-Light Emitting Diode (LED) irradiation is considered as an alternative to conventional UV lamps due to its low power consumption, small size, low toxicity and long shelf life [27]. According to Finardi et al. [28], this new source of UV light uses a semiconductor that allows light to be emitted at different wavelengths, and the first pasteurization studies performed were on water [29]. Although significant progress has been made, certain aspects of safety are still unclear.

On the other hand, it is known that fruit juices are very susceptible to contamination, mainly by pathogenic bacteria and some yeasts. Bacteria such as *Lactobacillus plantarum* are capable of impairing the taste of fruit juices [30]. In addition, *Escherichia coli* O157:H7 represents a current public health problem in several countries around the world [31]. UV light treatment is a technology that has been reported to inactivate pathogens in fruit juices [32,33].

Mathematical models have been used to evaluate the effect of UV light on pathogenic microorganisms such as *E. coli*, *Listeria innocua*, *Bacillus subtilis*, *Alicyclobacillus acidoterrestris*, *Pseudomonas aeruginosa*, *Legionella pneumophila*, mesophilic bacteria, and yeasts and molds in both water and liquid foods [13,34,35,36,37,38]. Based on the above, studies of the effect of UV-LED on fruit juices are needed. Among the studies that we highlight and that have maintained a microbiological focus on fruit and vegetable juices are Cheng et al. [39], Xiang et al. [40], Zhai et al. [41], D’Souza et al. [42] and Baykuş et al. [43]. Furthermore, a good efficiency of the UV-LED treatment can be optimized by means of a reactor design (static or dynamic) and by generating mathematical models. Mathematical modeling makes it possible to predict transport phenomena in food processing. This allows one to improve equipment design and process control management, and minimize excessive energy consumption and product damage [44,45].

UV-LED irradiation modeling on a liquid matrix is necessary in order to understand and predict the interaction of phenomena such as hydrodynamics and microbiological inactivation [46]. Soro et al. [47] explains that models are based on previous experimental data and parameters depend on factors such as time and the type of microorganism. Modeling the kinetics of inactivation can significantly reduce the number of tests required to determine the efficiency of a UV-C reactor [48]. However, recent studies mention that the UV-C LED irradiation process can be promising in the inactivation of pathogens; for this purpose, it is relevant to study the mathematical prediction of this process [49]. In addition, Atilgan et al. [48], Ghate et al. [35], Unluturk et al. [38], Baysal et al. [13] and Pratap-singh et al. [9] concluded that the effect of UV irradiation on the inactivation of pathogenic bacteria and spores was efficient and the experimental data were well fitted with the Weibull model. UV-C light has been approved for treating food surfaces and juices and has had FDA approval since 2004 [50]. Its application has been tested to reduce spoilage, microorganisms and pathogens in grape, carrot, orange and apple juices [13,51,52]. However, there are no studies of UV-LED processing on the inactivation of *Escherichia coli* O157:H7 and *Listeria monocytogenes* in tomato juice. Therefore, the main objective of this research was to evaluate the behavior of pathogenic bacteria *Escherichia coli* O157:H7 and *Listeria monocytogenes* inoculated in tomato juice (*Solanum lycopersicum*) processed with UV-LED irradiation to analyze the treatment’s effect on microbiological inactivation kinetics, Weibull distribution model and microbiological safety.

## 2. Material and Methods

### 2.1. Sample Preparation

**Figure 1:**
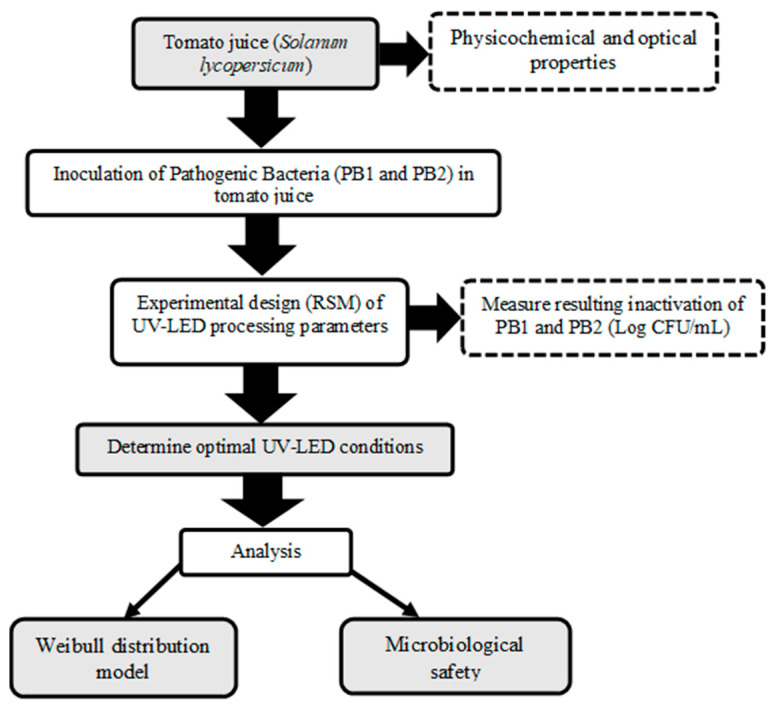
Experimental method scheme.

Organic tomato (*Solanum lycopersicum*) was obtained from Romeral, Valparaíso, Chile. The procedure for selecting, washing and cutting the samples was as follows: A commercial juicer (Panasonic MJ-70M) was used to separate the skin and seeds from the juice and then filter, centrifuge, remove solids and store at −80 °C until analysis. The control sample was heat-treated tomato juice. All details were adapted from Pizarro-Oteíza and Salazar [7]. The methodological scheme is shown in Figure 1.

### 2.2. Physicochemical and Optical Properties

Control samples were analyzed in triplicate for optical properties and physicochemical characterization in order to analyze the characteristics of unprocessed tomato juice. Samples were measured for pH soluble solid, color (CIE Lab), turbidity, viscosity and density, as proposed by Evangelista et al. [53], Mirondo et al. [54], and Pizarro-Oteíza and Salazar [7].

On the other hand, different dilution factors (2, 4, 8, 16, 32, 64 and 128) were used. The estimate of the absorption coefficient (cm^−1^) at wavelength was determined by plotting the absorbance against sample concentration proposed by Akgün and Ünlütürk [55].

### 2.3. Inoculation of Pathogenic Bacteria

Inoculations of pathogenic bacteria PB1: *Escherichia coli* O157:H7 (ATCC 2592) and PB2: *Listeria monocytogenes* (ATCC 19115) in tomato juice before treatments were obtained from the mother culture agar plates. For the subculture, a colony of the mother culture was suspended in broth and incubated at 37 °C for 48 h. Then, an aliquot of the subculture was transferred to a higher volume of media in a 1% (*v*/*v*) ratio to reach the desired final concentration of approximately 10^8^–10^9^ CFU/mL. This procedure was similar to that reported by Caminiti et al. [17] and Fredericks et al. [56]. PB1 and PB2 were cultured from lyophilized samples stored at −80 °C according to Akgün and Ünlütürk [55]. MacConkey Sorbitol (SMAC; Difco™, Grenoble, France) Agar and Oxford Agar base with antimicrobial supplement (OAB; MB Cell, Seoul, Republic of Korea) were used to selectively proliferate PB1 and PB2, respectively [57].

### 2.4. Evaluated Treatments

#### 2.4.1. UV-LED Irradiation

**Figure 2:**
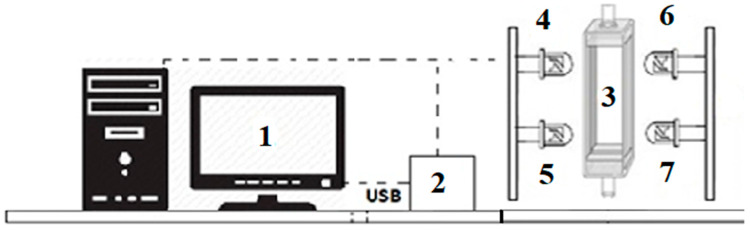
UV-LED irradiation processing. 1, Computer; 2, Power supply and control unit; 3, Irradiation zone and quartz cuvette; 4–7, UV-LED. Adapted from Pizarro-Oteíza and Salazar [7].

The effect of UV-LED irradiation on the microbiological activity of PB1 and PB2, inactivation kinetics and storage conditions were analyzed. The experimental method used a miniature system to replicate UV-LED irradiation in a static regime with a Spectrosil quartz cell (Starna Scientific Limited, Hainault, Essex, UK) placed vertically, according to a report by Pizarro-Oteíza and Salazar [7] (Figure 2). The volume (1.8 mL) and path length (1 mm) of the quartz cell were used. The average temperature of the UV-LED irradiation processing did not deviate from 28.5 ± 2 °C. The samples were treated with three wavelength diodes: 265 nm (Taoyuan Electron, Hong Kong, China), 272 nm (Taoyuan Electron, Hong Kong) and 278 nm (Vishay Semiconductors, Shanghai, China). In addition, the power intensities of these wavelengths were 2 mW, 3 mW and 10 mW, respectively.

#### 2.4.2. Heat Processing

Heat treatment of inoculated tomato juice was performed to compare with UV-LED treatment of inoculates. Heat treatment conditions were 70 °C for 14 min. Samples were agitated constantly in a thermoregulated bath with 2 mL of samples [7]. After heat treatment, bacterial counts of PB1 and PB2 were measured.

### 2.5. Experimental Design and Model Validation

Optimal conditions for UV-LED processing in the Box–Behnken design in two experimental blocks were determined with response surface methodology (RSM). The experimental design was statistically evaluated using Statgraphics Centurion XVI^®^ (Statpoint Technologies, Inc., Warrenton, VS, USA).

UV-LED process inputs were A: Power intensity (%), B: Time (min) and C: Wavelength (nm), and outputs were maximum inactivation of PB1 and PB2 (log CFU/mL). Levels for A, B and C were 50–70–90%, 7–14–21 min and 265–272–278 nm, respectively.

Furthermore, two experimental blocks (Run: 1–15 and Run: 16–30) were performed in duplicate to validate the design of the model and to obtain percent error with respect to the calculated value [58]. Verification of the optimal conditions calculated by RSM was carried out by testing the conditions experimentally and determining the absolute error “AE” calculated with Equation (1):

(1) $$AE\left(\%\right)=\left|\left.\frac{observed\;value-predicted\;value}{predicted\;value}\right|\times 100\right.$$

Response variables Y1: Inactivation log PB1, and Y2: Inactivation log PB2 were expressed individually as a second-order polynomial function with respect to the independent variables, as shown in Equation (2) [59,60].

(2) $$Y(i)=\beta_{0}+\sum_{i=1}^{k}\beta_{i}X_{i}+\sum_{i=1}^{k}\beta_{ii}X_{i}^{2}+\sum \sum \beta_{ij}X_{i}X_{j}$$

where Y(i): predicted response, β_0_: constant, β_i_: linear coefficient, β_ii_: quadratic coefficient, β_ij_: interaction coefficients, and X_i_ and X_j_: discrete levels of the independent variables.

Once UV-LED processing conditions were optimized, fluence was calculated (Equation (3)) as a function of UV-LED processing time [8,61,62].

(3) $$F=Fluence\left(\frac{mJ}{{cm}^{2}}\right)=Irradiance\left(\frac{mW}{{cm}^{2}}\right)\times time\left(s\right)$$

where irradiance (mW/cm^2^) is equal to UV intensity (mW) at a specific wavelength divided by the area of the quartz cell (18.1 cm^2^). Furthermore, the distance between the sample and the UV-LED source was 1 mm.

### 2.6. Weibull Distribution Model for Microbial Inactivation

Microbial inactivation of PB1 and PB2 was evaluated in treated tomato juice samples. Viable cell counts of PB1 and PB2 were analyzed and the log reduction (CFU/mL) calculated with respect to the initial load of the untreated sample. Once the optimal conditions for the UV-LED irradiation process were defined, the kinetics of microbiological inactivation were determined.

The non-linear Weibull distribution model (Equation (4)) analyzes the behavior of the microbial inactivation curve with a region of upward and downward concavity [48]. There are two kinetic parameters (p and δ) in the Weibull model [13,22].

(4) $$Y=\frac{N}{N_{0}}=e^{{-\left(\frac{F}{\delta }\right)}^{p}}$$

Survival curves were obtained by plotting the logarithm of the survival fractions (N/N_0_) versus the applied fluence UV-LED (F, mJ/cm^2^) on the tomato juice. N is PB1 and PB2 counts after UV-LED treatment and N_0_ is the initial number of PB1 and PB2 at inoculation. P determines the shape of the curve. If *p* > 1, concavity is downward; if *p* < 1, concavity is upward; and if *p* = 1, the model is linear, according to Atilgan et al. [48]. Δ is the scale parameter and is described as the UV necessary to obtain the first ten-fold reduction in the microbial population.

### 2.7. Microbiological Safety

The logarithmic count of pathogenic bacteria (PB1 and PB2) inoculated in tomato juice was analyzed to determine the storage conditions. The samples were treated with the optimal UV-LED conditions and thermal treatment. Microbiological counts were determined once incubation was completed, and storage conditions were analyzed for 28 days (0, 7, 14, 21 and 28) at 4.0 ± 0.82 °C in Eppendorf tubes [63]. Three different batches (*n* = 3) were considered separately to achieve statistical significance according to Briones et al. [64]. Furthermore, the control sample corresponded to day zero of storage.

### 2.8. Statistical Analysis

Means and standard deviations of the response surface methodology, Weibull modeling and storage conditions in tomato juice were obtained with a randomized ANOVA design using Statgraphics Centurion XVI^®^ software (Stat Point Technologies, Inc., Warrenton, VA, USA). In addition, Duncan’s multiple range test (MRT) was used to detect differences between means (*p* < 0.05). ANOVA was used to establish the statistical validation of the polynomial equations generated.

All the responses observed were concurrently fitted to different models. The best fitting experimental model was taken statistically on the basis of comparison of statistical parameter R^2^ and was carried out to measure the response (Yi). The fit quality of Weibull’s equation was evaluated using the Sum of Squared Errors (SSE), Chi-Squared Test (χ^2^) and Root-Mean-Square Error (RMSE), as shown in Equations (5)–(7) [11].

(5) $$SSE=\sum_{i=1}^{N}\frac{{\left(Y_{exp}-Y_{cal}\right)}^{2}}{N}$$

(6) $$\chi 2=\sum_{i=1}^{N}\frac{{\left(Y_{exp}-Y_{cal}\right)}^{2}}{N-m}$$

(7) $$RMSE=\sum_{i=1}^{N}\sqrt{\frac{{\left(Y_{exp}-Y_{cal}\right)}^{2}}{N}}$$

where Y_exp, or observed_ is the survival of the logarithmic count of PB1 and PB2 in tomato juice, Y_cal_ is the survival of the logarithmic count of PB1 and PB2 calculated by the Weibull model in tomato juice, N is data number or fluences, and m is the number of Weibull model constants.

## 3. Results and Discussion

### 3.1. Physicochemical Parameters and Optical Properties

The physicochemical and optical properties such as pH, soluble solids (°brix), turbidity (NTU), density (g/mL), viscosity (cP), chromatic coordinates (L, a* and b*) and absorption coefficient (cm^−1^) of the tomato juice were 4.02 ± 1.23, 4.8 ± 2.15, 6.35 ± 1.22, 1.11 ± 0.15, 0.93 ± 0.12, L: 89.23 ± 1.14, a*: −2.31 ± 0.25, b*: 1.57 ± 1.67, and 3.9 ± 0.12, respectively.

These parameters are of considerable importance because they are indicators of UV absorption in the sample and, therefore, provide information about the effect on microorganisms [22,65,66]. Authors have reported the similarity of some of these parameters in tomato and orange juices [6,22,67,68]. 

### 3.2. UV-LED Irradiation Process on the Response Variables

#### 3.2.1. Logarithmic Inactivation of PB1 and PB2

The Box–Behnken experimental design of the UV-LED irradiation process used the response variables of Y1 and Y2, where Y1: PB1 and Y2: PB2 were inactivated (Log CFU/mL), respectively; further, also used are factors such as A: power intensity (%), B: time (min) and C: wavelength (nm).

The values response variables for Y1 and Y2 were 0.02–3.14 and 0.14–2.73 (Log CFU/mL), respectively (Table 1). Table 2 shows the significant effects, regression parameters of the models, linear and quadratic terms, and factor interactions on the response variables (Y1 and Y2). Second-order polynomial models were observed for the response variables Y1 and Y2, and significant differences were observed in the models presented by Equations (8) and (9), respectively:

(8) $$Y1=-2\times 10^{-2}C^{2}-0.7\;A+1.43\;B+13\;C+3\times 10^{-3}AC-5\times 10^{-3}BC-1760$$

(9) $$Y2=1\times 10^{-3}A^{2}-1\times 10^{-2}C^{2}+0.16\;A+9\times 10^{-2}B+6.2\;C-1\times 10^{-3}AB\;-1\times 10^{-3}AC-858$$

The mathematical model of Y1 showed significant differences for the three factors of the experimental design—that is, A, B and C (*p* < 0.05). Also, quadratic factor (C^2^) together with the interactions AC and BC showed significant differences (*p* < 0.05). Furthermore, the value of R^2^ was 0.96 (Table 2), i.e., it generated a good fitness of the models with the experimental data.

**Figure 3:**
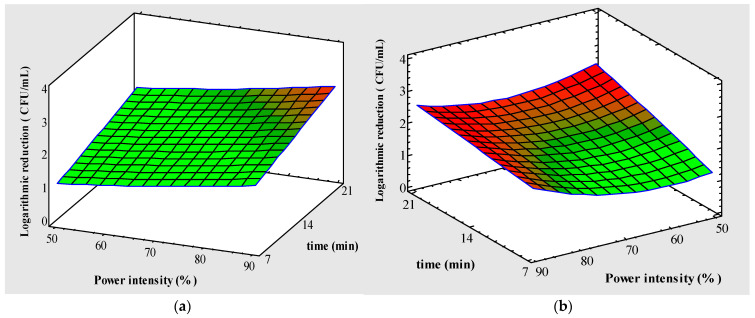
Effects of power intensity and time in UV-LED processing of tomato juice. (**a**) Inactivation of PB1 (Log CFU/mL) and (**b**) inactivation of PB2 (Log CFU/mL) at 272 nm. The red zone corresponds to a greater effect of the factors on the responses.

On the other hand, the polynomial response variable Y2 reported significant differences for the factors A, B and C with a good experimental setting (R^2^, 0.98) (Table 2). At the same time, the quadratic factors (A^2^ and C^2^) in conjunction with AB interaction and AC showed significant differences (*p <* 0.05). Additionally, the lowest value obtained from this response was in the A (50–70%), which could explain the behavior of the upward concavity curve, as observed in the positive quadratic factor (A^2^). This means that the response variable Y2 obtained minimum values in this condition of the experimental design, as shown in Figure 3b. On the other hand, substantial differences in the inactivation kinetics of PB1 and PB2 can be attributed different thermodynamic states and mobilities of *E. coli* and *Listeria* once subjected to UV-LED treatment [39]. Furthermore, UV sensitivities of Gram-positive bacteria, Gram-negative bacteria and yeasts differed from each other. For each microorganism groups, higher doses of irradiation resulted in higher reduction levels. Gram-negative organisms showed the lowest resistance while yeasts showed the highest resistance to UVC-LEDs [69]. There are few studies of the UV-LED process for response surface methodology (RSM) such as [70,71,72] and Park et al. [73], where different factors such as time, fluence, sample volume and power intensity, among others, were analyzed. However, we conclude that our design was significant and efficient for the logarithmic inactivation of PB1: *Escherichia coli* O157:H7 (Y1) and PB2: *Listeria monocytogenes* (Y2). Ultraviolet light focused with microbial inactivation is attributed to the absorption of ultraviolet light in the DNA of the microorganism, causing crosslinking between adjacent pyrimidine nucleotide bases. The formation of these dimers inhibits DNA replication and transcription and leads to cell death [74]. 

However, Bintsis et al. [14] reported that UV rays have a higher bactericidal potency depending on the type of liquid food. In addition, it has been thoroughly evaluated that not only is wavelength one of the critical factors but also others such as type of microorganism, impact on quality, inactivation mechanism, fluence and energy density [24]. In other words, to analyze an efficient UV-LED process on the inactivation of pathogenic bacteria, we must consider the above.

#### 3.2.2. Optimum Model Validation

UV-LED irradiation process conditions in the optimization by RSM and its maximized response variables of Y1 and Y2 were evaluated. The optimum results obtained for the three factors depend on the type of pathogenic microorganism [75]. Based on the above, if we analyze the Y1 and Y2 inactivation cases separately, they both provided different UV-LED irradiation process conditions because they represent a different polynomial. However, the optimizations of the Y1 and Y2 polynomials were similar in magnitude for factors A and B—90% and 21 min, respectively—and different with respect to parameter C, i.e., Y1 was 272.9 nm and Y2 was 275.4 nm. The above can be explained according to Kim et al. [69], who analyzed the bactericidal effect of UVC-LED (266 to 279 nm) for the inactivation of Gram-positive and Gram-negative foodborne pathogenic bacteria and yeasts. These authors showed that the membrane damage in all the microorganisms studied were different, but the greatest effect on inactivation was on DNA.

Additionally, the optimal conditions obtained from the factors were used to determine the validity of the prediction model used with respect to the experimental response variables and obtained an absolute error (AE) of 5.9% for inactivation of *Escherichia coli* O157:H7 (Y1) and 4.4% for inactivation of *Listeria monocytogenes* (Y2). Finally, according to the optimum conditions of the UV-LED irradiation process, the fluence was calculated with Equation (3) and an approximate value of 251 mJ/cm^2^ was obtained.

### 3.3. Microbial Inactivation Kinetics and Weibull Modeling

**Figure 4:**
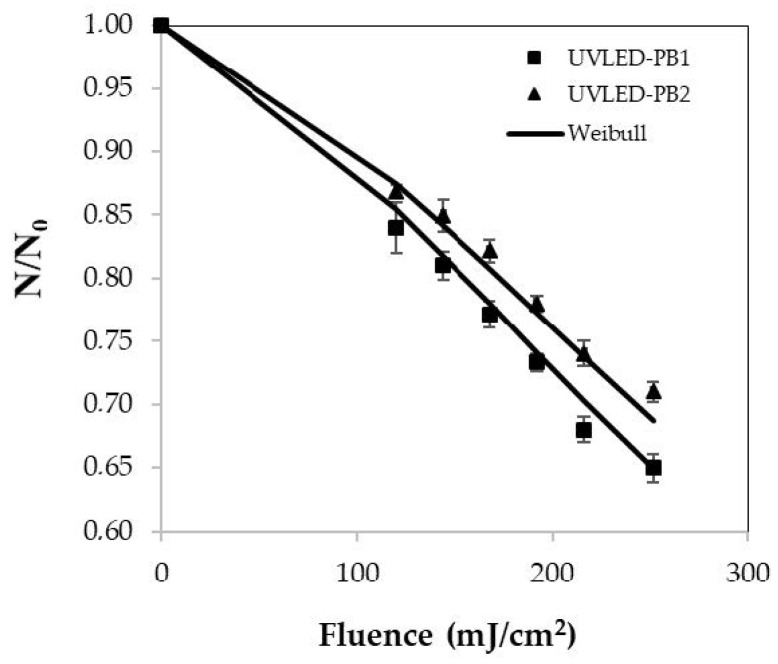
Survival fraction (N/N_0_) of inoculated tomato juice with PB1 and PB2 at different UV-LED fluences and modeled with Weibull.

The microbiological inactivation of pathogens PB1 and PB2 is shown in Figure 4. From the previously calculated fluences according to the optimum condition (251 mJ/cm^2^), fluences of 120, 144, 168, 192, 216 and 251 mJ/cm^2^ were also considered for the kinetics of microbial inactivation. These fluences have been reported in different fruit with log reduction in vegetable juices such as kale, grape and tomato; however, they gave a different behavior in the logarithmic inactivation [76,77,78]. The logarithmic kinetics of PB1 inactivation for the evaluated fluences of 120, 144, 168, 192, 216 and 251 mJ/cm^2^ were 1.62, 2.28, 2.48, 2.64, 2.78 and 3.1 (CFU/mL), respectively.

In turn, the logarithmic values of PB2 inactivation for 120, 144, 168, 192, 216 and 251 mJ/cm^2^ were 1.14, 1.50, 1.57, 2.14, 2.61 and 2.85 (CFU/mL), respectively. This shows that the higher the fluence, the greater the effect on the logarithmic inactivation of pathogenic PB1 bacteria: *Escherichia coli* O157:H7 and PB2: *Listeria monocytogenes*. Authors such as Unluturk et al. [38] have studied a similar behavior in liquid matrices of these pathogens exposed to UV-C radiation and Allahyari et al. [21] in water for inactivation of *Legionella pneumophila* and *Legionella dumoffii* exposed to UV-A-LED. Moreover, Ngadi et al. [78] studied apple juice and applied a UV fluence of 300 mJ/cm^2^ for 16 min to achieve a logarithmic reduction of 4.2 CFU/mL in *E. coli* O157:H7 ATCC 35150. Zhai et al. [41] reported that UV-C-LED irradiation reduced the population of *Zygosaccharomyces rouxii* in apple juice at 4.86 log CFU/mL at 800 mJ/cm^2^. These authors concluded that the treatment has potential application in the juice processing industry. Akgün and Ünlütürk [55] evaluated UV fluence of 707.19 mJ/cm^2^ for 40 min in apple juice and generated of 3.70 CFU/mL of *E. coli* K12 using 280 and 280/365 nm UV-LED combinations. This provides important information to produce a more significant effect in the inactivation of pathogenic bacteria. In addition, Baykuş et al. [43] reported the efficacy of UV-LED with wavelengths of 280, 365 and 280/365 nm in the inactivation of *E. coli* K12 in mixed fruit drinks and concluded that UV at 280 and 280/365 nm generated more log reductions than at 365 nm. Green et al. [79] evidenced the combination of 259 and 289 nm wavelengths to be an efficient germicidal combination against certain microorganisms for UV-LED. Based on the above, the correct combination of defined wavelengths, time and power intensity, and characterization of physicochemical and optical properties could increase the logarithmic reduction in pathogenic bacteria PB1: *Escherichia coli* O157:H7 and PB2: *Listeria monocytogenes* in tomato juice.

Kinetic models allow predicting microbial behavior under specific environmental conditions, optimizing the process and inactivation rates to significantly reduce the amount of testing required [48,78,80]. In addition, they can help quantify bacterial growth as a function of intrinsic process effects such as light intensity and extrinsic effects such as the location of the liquid in the reactor [48]. Figure 4 represents the survival fraction (N/N_0_) of inoculated tomato juice with PB1 and PB2 at different UV-LED fluences and fitted with the Weibull model. Application of the Weibull model to the experimental data was analyzed by linearizing the double logarithms ln (−ln(N/N_0_)) versus ln (F) to calculate the kinetic parameters [38].

Scale parameter (p) was measured to determine the concavity index (Table 3). The values for PB1 and PB2 were 1.37 ± 0.1 and 1.40 ± 0.1, respectively. This indicates that the remaining cells are more likely to become less and less resistant to the higher fluence evaluated [7,30,68].

Furthermore, Atilgan et al. [48] mentioned that a concave downward curve exists when *p* > 1, which can be seen in Figure 4. The parameter δ, which according to Taze et al. [22] and Baysal et al. [13] is the decimal reduction (D10) time or fluence required for a 90% of log microorganisms inactivation, obtained values of 465.2 ± 37.5 and 511.3 ± 47.6 (mJ/cm^2^) for PB1 and PB2, respectively. Also, the scale parameter δ is often considered a measure of the organism’s resistance (kinetic parameter) to treatment [38]. Based on the above, we can conclude that 465.2 mJ/cm^2^ is needed to achieve 90% log reduction in PB1 and 511.3 mJ/cm^2^ for PB2. According to the results, the D10 or δ value of this study is higher than those indicated in the bibliography [81].

Tran and Farid [82] reported that δ was 119 ± 17 mJ/cm^2^ for yeasts and molds in orange juice. Torkamani and Niakousari [83] showed a D10 value in orange juice of 105 mJ/cm^2^. Zhai et al. [41] obtained an average value of 56 (mJ/cm^2^) on the inactivation of *Alicyclobacillus acidoterrestris* in orange juice, and Baysal et al. [13] calculated a value of 9.55 (mJ/cm^2^) on the inactivity of *Alicyclobacillus acidoterrestris* in apple juice, among others. On the other hand, Table 3 presents the goodness of fit in terms of R^2^. The Weibull model fitted the experimental data of the tomato juice better, as indicated by the lower RMSE, SSE and χ^2^ values.

### 3.4. Microbiological Safety

The bacterial load of inoculated tomato juice processed with UV-LED irradiation and heat treatment was analyzed. For this, PB1 and PB2 pathogenic bacteria were counted on different days of storage. PB1 and PB2 counts (log CFU/mL) increased during post-treatment days compared to zero day (control). PB1 and PB2 counts for the UV-LED treatment on the zero-storage day were 5.55 ± 0.03 and 5.30 ± 0.17, respectively.

**Figure 5:**
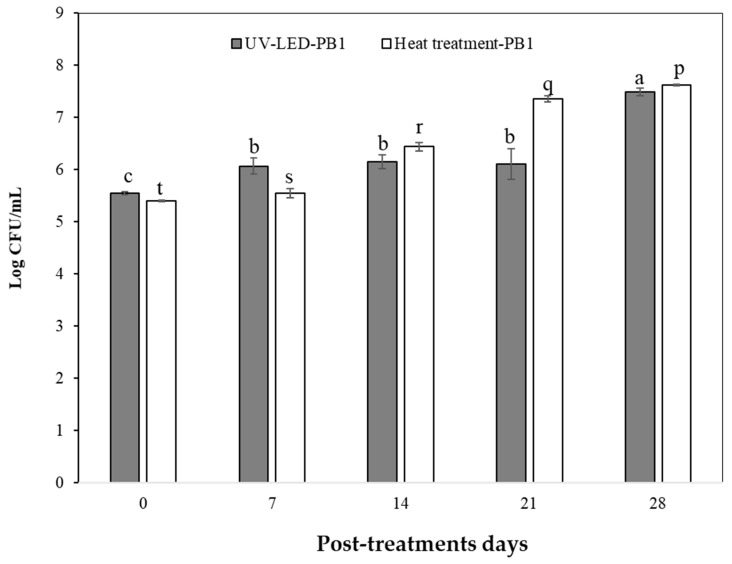
PB1 count (Log CFU/mL) inoculated in tomato juice processed with UV-LED irradiation and heat treatment. Letters (a, b and c) in each bar represent significant differences with respect to storage days (0, 7, 14, 21 and 28) for UV-LED treatment. Letters (p, q, r, s and t) in each bar represent significant differences with respect to storage days for heat treatment.

**Figure 6:**
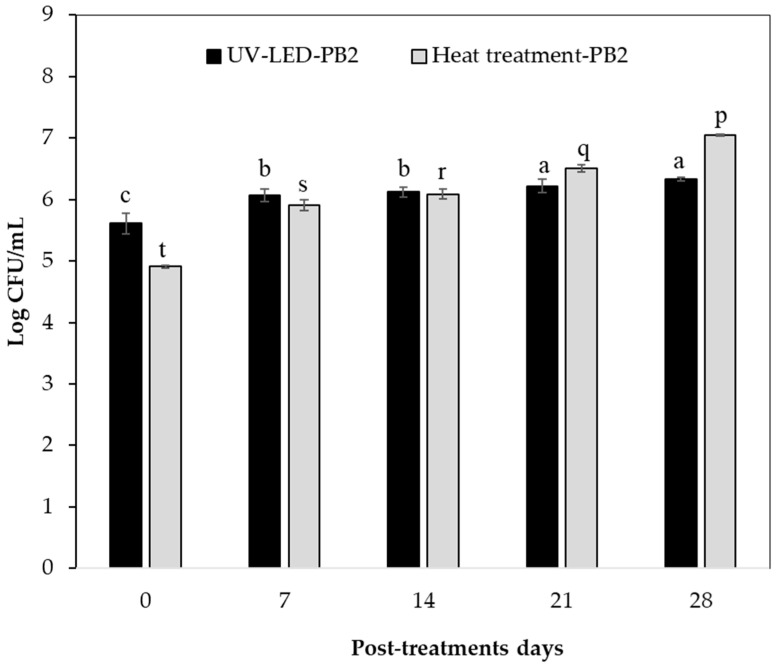
PB2 count (Log CFU/mL) inoculated in tomato juice processed with UV-LED irradiation and heat treatment. Letters (a, b and c) in each bar represent significant differences with respect to storage days (0, 7, 14, 21 and 28) for UV-LED treatment. Letters (p, q, r, s and t) in each bar represent significant differences with respect to storage days for heat treatment.

The UV-LED treatment evidenced a constant PB1 count (log CFU/mL) on days 7–14 and 21 (Figure 5). The opposite occurred on day 28, as an increase in the count of this pathogen was observed (*p <* 0.05). This could be an indicator of UV-LED technology having an antibacterial effect on PB1 counts (log CFU/mL) up to day 21 at 4 °C. Furthermore, the increase in PB1 count (log CFU/mL) at day 28 is due to cells being exposed to sublethal damage and, in some cases, recovering after treatment during the storage period [84]. In relation to the UV-LED treatment, the PB2 count (log CFU/mL) remained constant on days 7 and 14, and increased on days 21 and 28, but showed no significant differences (*p* > 0.05). The foregoing can provide information on the antibacterial effect of UV-LED technology and shows that its microbiological safety was maintained for up to 14 days of storage at 4 °C (Figure 6). Based on the above, more studies are needed to clarify this effect applied in such technology.

Among the studies to highlight, mainly linked to UV technology applications on the inactivation of foodborne pathogens, Fan et al. [85] investigated the effect of UVC-LEDs (275 nm) on the inactivation of foodborne pathogens of raw tuna fillets. Raw tuna fillets inoculated with cocktails of *Salmonella typhimurium*, *Listeria monocytogenes* and *Escherichia coli* O157:H7 were exposed to UVC-LEDs at 500–4000 mJ/cm^2^. UVC-LED treatments of single-layer tuna fillets at a dose of 4000 mJ/cm^2^ resulted in 1.31–1.86-, and 1.77- log reduction in *S. typhimurium*, *L. monocytogenes* and *E. coli* O157:H7, respectively. Nevertheless, Taze et al. [22] reported that strategies should be evaluated to improve the effectiveness of UV in inactivating microorganisms and consequently improve shelf life. Another work by Chun et al. [86] evidenced that the efficacy of UV-C radiation to inactivate *E. coli* O157:H7 and *Listeria monocytogenes* on fresh-cut salad increased with increasing UV dose from 100 to 800 mJ/cm^2^. UV doses of 800 mJ/cm^2^ reduced *E. coli* and *L. monocytogenes* counts on fresh-cut salad by 2.16 and 2.57 log CFU/g, respectively. Furthermore, Lim et al. [87] studied the efficacy of UV-C irradiation (0 to 223.1 mJ/cm^2^) to reduce *Salmonella* contamination at various locations on green tomatoes. They reported that regardless of the location of the tomatoes, UV-C treatment was shown to be effective in reducing the *Salmonella* levels. Based on the above, UV sensitivity of microorganisms varies between species to species significantly due to the differences in cellular components such as cell wall composition (thickness and Gram-type), structure of nucleic acid (Pyrimidine G+C content), type of cellular proteins, photoproducts, physiological state of microorganism and the ability of the cell to repair UV damage [18]. In addition, the efficacy of UV treatment may vary by growth media, stage of culture, density of organisms and surface characteristic of the food [15]. It is worth noting that poor penetration power, irregular dose delivery and long treatment times are major limitations of UV treatment applications on the inactivation of foodborne pathogens [15]. On the other hand, the PB2 count (log CFU/mL) on storage day zero (control) with heat treatment was lower than that of UV-LED, by about 14%, and the PB1 count was approximately 2.7% lower. In addition, on day 28 of storage, the PB2 count of tomato juice treated with UV-LED was 11.4% less than the heat treatment mentioned above. That is to say, this technology could be an alternative method to heat treatment to preserve the PB2 count in tomato juice up to 28 post-treatment days at 4 °C; however, a higher antibacterial effect is needed, and this could be improved with higher fluences, combined treatments or other operating conditions to achieve an increase in the inactivation of foodborne pathogens in fruit juices. This is mainly because, to make a fruit juice fit for consumption, the reduction in bacterial population with treatment should be about 5 log [74].

## 4. Conclusions

The present study confirms that UV-LED irradiation is efficient for inactivating pathogenic bacteria (PB1: *Escherichia coli* O157:H7 and PB2: *Listeria monocytogenes*), and this process could be optimized using the RSM Box–Behnken design. The optimal conditions of A: power intensity (%), B: time (min) and C: wavelength (nm) were 90%, 21 min and 273–275 nm, respectively, resulting in a fluence of approximately 251 mJ/cm^2^. The proposed model shows the quantitative effect of the maximized Y1 and Y2 response variables and the linear and quadratic interactions of the factors. Based on the optimal conditions, the log inactivation of PB1 (Y1) was 2.89 (CFU/mL) with a 5.9% absolute error (AE) and the log inactivation of PB2 (Y2) was 2.74 (CFU/mL) with a 4.4% AE. The Weibull model obtained a good fit to the experimental data of PB1 and PB2 log inactivation with R^2^, SSE, χ^2^ and RMSE values of 0.91, 0.0002, 0.0001 and 0.0113, respectively. In addition, the kinetic parameter δ showed that 465.2 mJ/cm^2^ is needed to achieve a 90% log reduction in PB1 and 511.3 mJ/cm^2^ for PB2 (CFU/mL). In addition, the log survival of PB1 *Escherichia coli* O157:H7 and PB2: *Listeria monocytogenes* increased as the days of storage increased; however, PB2 in juice treated with UV-LED contained 11.4% less bacteria than heat treatment on the 28th day and maintained a similar count (log CFU/mL) on days 21–28, which could be efficient to use as a method of preserving tomato juice until day 21. Finally, heat treatment has been the method of preservation in fruit and vegetable juices; nevertheless, there is a high probability that they may lose sensory and nutritional quality. Moreover, consumers are increasingly relying on this rationale when consuming a product and are looking for an alternative to conventional treatments. Therefore, studies are needed to replicate a larger scale of the experimental design and clarify the effect of UV-LED technology on the inactivation of pathogenic bacteria in other fruit- and vegetable-based liquid foods.

## Figures and Tables

**Table 1:** Box–Behnken design to UV-LED irradiation conditions and experimental or observed inactivation of PB1 and PB2 (Log CFU/mL). Two experimental blocks (Runs 1–15 and Runs 16–30).

Run	UV-LED Irradiation Conditions			Inactivation PB1 (Log CFU/mL)	Inactivation PB2 (Log CFU/mL)
	A	B	C	Observed	Observed
1	90	7	272	1.86	2.19
2	50	14	278	0.75	2.39
3	70	7	265	0.02	0.14
4	70	7	278	1.18	1.45
5	50	7	272	0.84	1.07
6	90	21	272	3.14	2.73
7	50	14	265	0.60	0.72
8	50	21	272	2.00	2.42
9	70	14	272	1.90	1.71
10	70	21	265	1.05	0.95
11	90	14	278	1.96	2.32
12	90	14	265	0.51	1.19
13	70	14	272	1.89	1.71
14	70	21	278	1.30	2.31
15	70	14	272	1.91	1.70
16	90	7	272	1.85	2.19
17	50	14	278	0.68	2.35
18	70	7	265	0.03	0.15
19	70	7	278	1.19	1.46
20	50	7	272	0.83	1.10
21	90	21	272	3.12	2.71
22	50	14	265	0.58	0.71
23	50	21	272	2.00	2.42
24	70	14	272	1.90	1.69
25	70	21	265	1.02	0.96
26	90	14	278	1.93	2.31
27	90	14	265	0.49	1.20
28	70	14	272	1.89	1.69
29	70	21	278	1.24	2.32
30	70	14	272	1.88	1.68

**Table 2:** Effect of independent variables of UV-LED irradiation on the evaluated response variables. Behavior of the regression coefficients (β), R^2^ and significant factors of the design.

Response	Inactivation PB1 (Log CFU/mL)		Inactivation PB2 (Log CFU/mL)	
Factor	Regression Coefficient (β)	*p*-Value	Regression Coefficient (β)	*p*-Value
A: Power intensity (%)	−0.6921	0.000 *	0.1642	0.000 *
B: Time (min)	1.4288	0.000 *	0.0961	0.000 *
C: Wavelength (nm)	13.0194	0.000 *	6.1759	0.000 *
A^2^	0.00015	0.4171	0.0010	0.000 *
AB	0.00019	0.6937	−0.0014	0.000 *
AC	0.00250	0.000 *	−0.00103	0.000 *
B^2^	0.00001	0.9931	−0.00024	0.7798
BC	−0.00508	0.000 *	0.00027	0.7634
C^2^	−0.02407	0.000 *	−0.01115	0.000 *
R^2^	0.9597		0.9833	
R^2^_adj_	0.9386		0.9745	
MAPE	0.1249		0.070	
DW	2.070		1.710	

*: *p* < 0.05.

**Table 3:** Parameters and statistical indices of the Weibull model for pathogenic bacteria (PB1 and PB2).

Pathogenic Bacteria (PB)	Parameters		Statistical Indices		
	δ (mJ/cm^2^)	p	r^2^	SSE	RMSE
PB1: *Escherichia coli* O157:H7 (ATCC 2592)	465.2 ± 37.5	1.37 ± 0.1	0.9	0.0001	0.0115
PB2: *Listeria monocytogenes* (ATCC 19115)	511.3 ± 47.6	1.40 ± 0.1	0.9	0.0001	0.0110

## Data Availability

The data presented in this study are available on request from the corresponding author. The data are not publicly available because the data are confidential.
